# Melodia: a Python library for protein structure analysis

**DOI:** 10.1093/bioinformatics/btae468

**Published:** 2024-07-22

**Authors:** Rinaldo W Montalvão, William R Pitt, Vitor B Pinheiro, Tom L Blundell

**Affiliations:** Rega Insitute for Medical Research, Department of Pharmaceutical and Pharmacological Sciences, Herestraat 49, Leuven 3000, Belgium; Evotec (UK), 95 Park Drive, Milton Park, Abingdon, Oxfordshire OX14 4RY, United Kingdom; Rega Insitute for Medical Research, Department of Pharmaceutical and Pharmacological Sciences, Herestraat 49, Leuven 3000, Belgium; Heart and Lung Research Institute, University of Cambridge, Biomedical Campus, Trumpington, Cambridgeshire CB2 0BB, United Kingdom

## Abstract

**Summary:**

Analysing protein structure similarities is an important step in protein engineering and drug discovery. Methodologies that are more advanced than simple RMSD are available but often require extensive mathematical or computational knowledge for implementation. Grouping and optimizing such tools in an efficient open-source library increases accessibility and encourages the adoption of more advanced metrics. Melodia is a Python library with a complete set of components devised for describing, comparing and analysing the shape of protein structures using differential geometry of 3D curves and knot theory. It can generate robust geometric descriptors for thousands of shapes in just a few minutes. Those descriptors are more sensitive to structural feature variation than RMSD deviation. Melodia also incorporates sequence structural annotation and 3D visualizations.

**Availability and implementation:**

Melodia is an open-source Python library freely available on https://github.com/rwmontalvao/Melodia_py, along with interactive Jupyter Notebook tutorials.

## 1 Introduction

Analysing the protein backbone similarities allows us to gain insights into the relationship between structure and function in biochemistry. This knowledge contributes to structural biology, bioinformatics, and drug discovery, where a detailed understanding of protein structures is crucial for designing therapeutics and understanding cellular processes. However, comparing the geometry of protein backbones using their atomic coordinates can be challenging. The successful identification of structurally conserved regions, analysis of conformal dynamics changes during molecular dynamics, and the impact of amino-acid mutations in the conformational ensemble are a few examples of geometric analysis that demand a measure for structural divergence between two protein fragments.

Most current measures, such as RMSD, violate the triangle inequality rule and cannot judge dissimilarity when significant structural divergences are at play (Røgen and Fain 2002). Differential geometry, a branch of mathematics that deals with the geometry of smooth curves and surfaces, is a robust tool for the mathematical analysis of the 3D structure of a protein backbone as defined by a curve joining all its C*α* atoms. In Melodia, we use it to overcome the limitations of RMSD, such as superposition dependence, lack of sensitivity to minor structural changes, and the inability to measure the dissimilarity between structures. The differential geometry descriptors (curvature and torsion) are rotational and translational invariant, can sense even small conformational changes, and satisfy the triangle inequality rule, thus allowing their use on clustering algorithms to identify structurally conserved regions in protein families with significant structural divergences.

Rackovsky and Scheraga conducted early attempts to develop a differential geometry representation of protein backbones. They showed that a geometry representation using curvature and torsion can quantitatively compare the local folding of backbone structures, inspect the initial stages of protein folding, and predict which structures are likely to be formed. These results are possible because their differential geometry representations operate on a four-C*α* length scale, highlighting structural features not visible in by the *ϕ*/*ψ* dihedral angles as they operate on a single-residue length scale (Rackovsky and Scheraga 1978, 1980, 1981, 1982, 1984, Rackovsky and Goldstein 1987). Louie and Somorjai used differential geometry to study protein structural and dynamic patterns (Louie and Somorjai 1982, Louie *et al.* 1983). Their analysis suggested that the Frenet-Serret frame, a mathematical framework used in differential geometry to describe the local geometric properties of a curve or trajectory in 3D space, is the most suitable structure for a ‘unifying and natural description of the 3D conformation of proteins’. The works by Røgen and Bohr (Røgen and Bohr 2002) and Hu (2013) further extend the details of the theory of differential geometry applied to the protein backbone. In particular, Hu’s work presents an innovative and impressive algorithm for loop closure.

We have successfully used differential geometry to build bioinformatics tools for many years. CHORAL (Montalvao *et al.* 2005) was our first application of curvature and torsion descriptors for analysing structural similarities in protein families. It utilizes a Machine Learning method that uses differential geometry for pattern recognition to identify conserved structural patterns in homologous protein families. In addition, it uses environment-specific propensity tables (Deane and Blundell 2001) to classify and select patterns that most likely represent the core structure of a target protein. In our benchmarks, CHORAL produces models equivalent to that of MODELLER (Webb and Sali 2017), but in a fraction of the time. Polyphony (Pitt *et al.* 2014) is another application in which we have utilized differential geometry as its base for the analysis of multiple structures of a protein. It uses statistical approaches that rely directly upon residue equivalence rather than superposition. It can identify hinge regions, allosteric conformational changes and transient binding sites, and it is a helpful tool for rational drug design.

Our most recent method, FleXgeo (da Silva Neto *et al.* 2019) utilizes differential geometry representation in the analyses of protein conformational ensembles, focusing on the ones generated through Molecular Dynamics. It uses a new dissimilarity for protein flexibility measurement and a local conformational clustering method. Its measurement presents equally excellent or superior results compared to RMSF, especially for the intrinsically unstructured protein. The clustering method is unique as it relates protein global to local dynamics by providing global clustering solutions per residue, and it has many possible applications (da Silva Neto *et al.* 2020, Bertelli *et al.* 2021).

The popularization of AI methods, particularly Deep Learning, has opened several new possibilities for applications of differential geometry for protein bioinformatics. One of the recent applications of differential geometry in Deep Learning is SSnet (Verma *et al.* 2021), a very successful method for Protein–Ligand Interaction prediction. It encodes the information about the secondary structure of the proteins using the curvature and torsion of their backbones. The Neural Network uses this information to predict the interaction in combination with the ligand descriptors.

The SSnet results inspired us to release the Melodia library as open-source to allow easy access to differential geometry descriptors in a modern framework. Melodia focuses on two principles: (i) standardize the mathematical model to avoid differences in curvature and torsion values due to the different choices for backbone representation, and (ii) work with popular bioinformatics and machine learning tools.

## 2 Materials and methods

### 2.1 Mathematical approach

We apply *cubic spline interpolations* to fit the C*α* atoms to produce parametric curves of the backbone structure, thus creating smooth, continuous curves representing the protein topology. We fit the atomic coordinates into three parametric equations, with the residue number as their parameter. The result is a parametric vector Equation (1) where each parametric equation, for the individual coordinates, is a spline function instead of an analytical function.

(1) $$\vec{r}\left(t\right)=x\left(t\right)\hat{x}+y\left(t\right)\hat{y}+z\left(t\right)\hat{z}$$

The parametric spline representation preserves all the essential structural features in the secondary structure, such as *α*-helices and *β*-strands. The following equations compute curvature (2) and torsion (3) for the 3D curve at each C*α* parametric position (*t*).

(2) $$\kappa =\frac{\left|\dot{r}\times \ddot{r}\right|}{{\left|\dot{r}\right|}^{3}}$$

(3) $$\tau =\frac{\left(\dot{r}\times \ddot{r}\right)\cdot \dddot{r}}{{\left|\dot{r}\times \ddot{r}\right|}^{2}}$$

Although a bicubic spline representation fully characterizes the protein backbone geometry, more is needed for the numerical evaluation of the derivatives used by Equations (2) and (3). As the splines depend on third-order polynomials, the second and third numerical derivatives on those equations cause instabilities for a direct calculation from Equation (1). In Melodia, we use the cubic spline parametric functions as a framework for enforcing the characteristics we want into the backbone shape, and the derivatives are evaluated by using a *Chebyshev approximation* around the residue (Broucke 1973). At residue *i*, the parametric function is approximated by the *Chebyshev* function using fifty points from *i *−* *1 to *i *+* *1, and the coefficients are exploited to evaluate all the derivatives. This combination ensures a near-perfect modelling of the backbone curve and a fast and stable numeric computation of the differential geometry values. [see Montalvao *et al.* (2005), Leung *et al.* (2012), and Pitt *et al.* (2014) for additional details on this calculations]

It is easy to observe that the values computed through Equations (2) and (3) are very sensitive to the shape of the spatial curve described by Equation (1). Equation (1) accepts different choices for the bicubic splines implementation; each one will produce slightly different shapes with minor differences in curvature and torsion. Although there is no *right way* of selecting a space curve to represent the protein backbone, by choosing the one selected for Melodia, we can have consistent results in our applications, assuring the reproducibility of Machine Learning experiments. References Røgen and Bohr (2002) and Guo and Cremer (2016) scrutinize the impact on the differential geometry by the choice of representation for the curve. In particular, reference Guo and Cremer (2016) clarifies the reason for choosing the cubic spline as the most satisfactory representation.

Melodia also implements a knot theory descriptor, the writhing number (Chang *et al.* 2006), used to characterize a five-residue-long region around a central residue. This number is a geometric measure that describes the degree of curvature of the protein backbone formed from the vectors connecting all the C*α* atoms in that extended region. Although this measure describes a type of curvature for the protein backbone, it differs significantly from the curvature depicted by differential geometry. Differential geometry curvature is always positive (representing the deviation from a straight line). It conveys the local degree of curvature in space. The writhing number can be considered a type of *pseudo-curvature* as it depicts a large region and can show positive or negative values.

The library also provides access to environment-specific propensity tables (Deane and Blundell 2001) that can be used for generating Probability Density Functions (PDFs) for restraint-based conformational sampling (Montalvao *et al.* 2005, Leung *et al.* 2012). As demonstrated in our previous works, it has excellent potential for Deep Learning applications for protein structural modelling and design. The differential geometry curvature and torsion are high-accuracy descriptors of conformal changes in the backbone geometry and an exceptional source of information for Neural Network training.

### 2.2 Implementation

**Figure 1.**
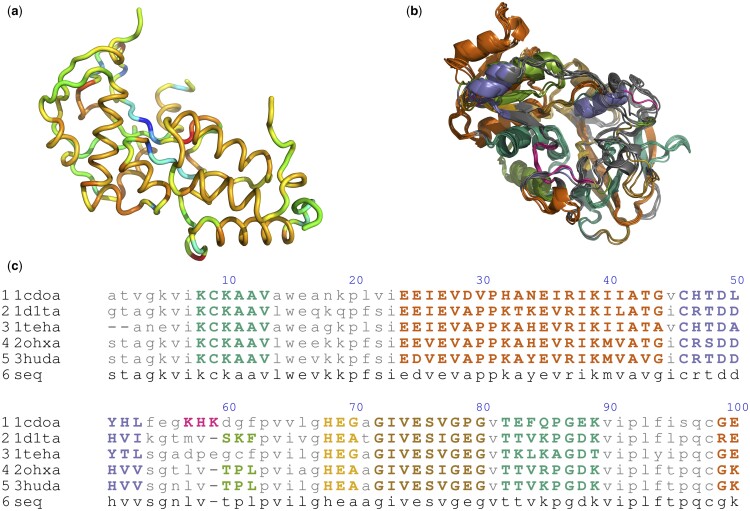
Examples of Melodia applications: (a) Protein (2k5x) backbone representation using a tube modelled over the C*α* spline fitting. The colours represent the values of the local curvature. (b) PyMol view of the alcohol dehydrogenase protein family, colour-coded by geometric similarity. (c) Segment of the alcohol dehydrogenase sequence colour-coded by geometric similarity. Melodia uses the same colour for both (b) and (c) visualizations.

Melodia is implemented in Python using the recommended software development best practices (Mayer 2022) and the Python code style (https://peps.python.org/pep-0008). All the arguments in the functions are annotated with type hints and thoroughly documented. We utilize the structure object from BioPython (Cock *et al.* 2009) for PDB parsing and data storage. BioPython is one of the most used open-source Python tools and the *de facto* standard for protein bioinformatics. Melodia can store its descriptors, or user-defined ones, as *b-factors* and output them for visualization with PyMol (http://www.pymol.org/pymol). Figure 1a shows an example of PyMol visualization of the tube cartoon model coloured by *b-factor*, whose values correspond to the backbone local curvature. Melodia also utilizes BioPython for sequence alignment storage and annotation, and it can output the alignment as a colour-coded Post-Script file and a PyMol script for superposing all the proteins in it. Figure 1b and c shows the results of the sequence annotation using Melodia’s algorithm for clustering protein blocks of equivalent geometry. Melodia is configurable and easily adjusted to match any user-defined protein local similarity criterion.

Our previous applications used the Numerical Recipes (Press *et al.* 1992) algorithms for cubic splines and *Chebyshev* interpolation. As its license is very restrictive and incompatible with our open-source goal, we adopted the NumPy (Harris *et al.* 2020) and SciPy (Virtanen *et al.* 2020) versions of those functions. They are high-performance, well-maintained, and robust code that many users have adopted and tested. By utilizing these libraries, we ensure the homogeneous behaviour of Equation (1) across all Operating Systems and Python versions. In addition, the standardization of the numerical methods guarantees that the curvature and torsion will be the same in all foreseeable situations.

Melodia implements lightweight data storage using Python dictionaries and the more advanced DataFrame object from the Pandas library (https://pandas.pydata.org/). The Pandas library DataFrame is compatible with popular data science libraries, like scikit-learn (Pedregosa *et al.* 2011), PyTorch (Paszke *et al.* 2019), and TensorFlow (Abadi *et al.* 2016), for example. It also works well with Jupyter Notebooks (Kluyver *et al.* 2016), supporting 3D visualization of the annotated protein backbone using nglview (Nguyen *et al.* 2018). All Melodia features are documented in Notebook tutorials (Supplementary Appendix), covering examples ranging from primary usage to more advanced structural analysis. An extensive study regarding this methodology can be found in Guo and Cremer (2016).

## 3 Conclusion

We present Melodia, a Python library designed to provide a robust and convenient protein structural analysis and visualization method using differential geometry and knot theory. Many bioinformatics applications have successfully used this method, demonstrating its excellence in describing protein structural patterns compared to other traditional approaches. Melodia introduces a standard and easy-to-install open-source code for use with Machine and Deep learning frameworks. Its data models are fully compatible with the most used Python libraries in bioinformatics and data science.

## Supplementary Material

btae468_Supplementary_Data

## Data Availability

The source code and data are available at https://github.com/rwmontalvao/Melodia_py.
