# Supplementary material for: Melodia: a Python library for protein structure analysis
Source: Bioinformatics. 2024 Jul 22;40(7):btae468. doi: 10.1093/bioinformatics/btae468 (PMC11290362; doi:10.1093/bioinformatics/btae468)
Supplement: btae468_Supplementary_Data [file btae468_supplementary_data.pdf]

Appendix A – Tutorial Notebooks

1 – Getting Started

In this notebook, we provide documentation for all the basic functions of Melodia. It has examples of compatibility with Biopython's PDB classes, Pandas DataFrames, Scikit-Learn, and NGLView. The notebook illustrates the data loading process from different sources and multiple data visualizations in 2D and 3D.

2 – Alignment Basics

This notebook documents the functions that works with protein sequence alignments and analyse structural similarities in a protein family. It shows how Machine learning clustering algorithms are used to determine structural conservation among family members. It also shows how the function for outputting PyMol scripts for visualizing the conservation operates.

3 – Basic Similarity Analysis

In this notebook, we delve deeper into the automatic analysis of protein similarity using differential geometry. It demonstrates the curvature and torsion sensitivity to even the smallest changes in the protein backbone geometry and contrasts the differential geometry approach with the traditional RMSD method.

4 – Advanced Similarity Analysis

This advanced example illustrates how to manually use Melodia to compare structural similarities between protein family members. It also shows how to use curvature and torsion to construct robust descriptors for finding protein family building blocks.

5 – Machine Learning Ensemble Analysis

In this notebook, we employ Machine Learning methods to analyse the structural variations (flexibility) in a protein. This is a straightforward illustration of how differential geometry can be used to classify protein backbone plasticity.

6 – Alignment Clustering and PDB Superimposition

This example illustrates how to use Melodia to compare structural similarities between protein family members and superimpose their structures. It employs a clustering algorithm to determine all the highly conserved regions across a family of proteins and a Simulated Annealing procedure to minimize their superimposition.
